# Carotenoids Play a Positive Role in the Degradation of Heterocycles by *Sphingobium yanoikuyae*

**DOI:** 10.1371/journal.pone.0039522

**Published:** 2012-06-20

**Authors:** Xiaorui Liu, Zhonghui Gai, Fei Tao, Hongzhi Tang, Ping Xu

**Affiliations:** State Key Laboratory of Microbial Metabolism & School of Life Sciences and Biotechnology, Shanghai Jiao Tong University, Shanghai, People′s Republic of China; Belgian Nuclear Research Centre SCK/CEN, Belgium

## Abstract

**Background:**

Microbial oxidative degradation is a potential way of removing pollutants such as heterocycles from the environment. During this process, reactive oxygen species or other oxidants are inevitably produced, and may cause damage to DNA, proteins, and membranes, thereby decreasing the degradation rate. Carotenoids can serve as membrane-integrated antioxidants, protecting cells from oxidative stress.

**Findings:**

Several genes involved in the carotenoid biosynthetic pathway were cloned and characterized from a carbazole-degrading bacterium *Sphingobium yanoikuyae* XLDN2-5. In addition, a yellow-pigmented carotenoid synthesized by strain XLDN2-5 was identified as zeaxanthin that was synthesized from *β*-carotene through *β*-cryptoxanthin. The amounts of zeaxanthin and hydrogen peroxide produced were significantly and simultaneously enhanced during the biodegradation of heterocycles (carbazole < carbazole + benzothiophene < carbazole + dibenzothiophene). These higher production levels were consistent with the transcriptional increase of the gene encoding phytoene desaturase, one of the key enzymes for carotenoid biosynthesis.

**Conclusions/Significance:**

*Sphingobium yanoikuyae* XLDN2-5 can enhance the synthesis of zeaxanthin, one of the carotenoids, which may modulate membrane fluidity and defense against intracellular oxidative stress. To our knowledge, this is the first report on the positive role of carotenoids in the biodegradation of heterocycles, while elucidating the carotenoid biosynthetic pathway in the *Sphingobium* genus.

## Introduction

Bioremediation is an environment-friendly alternative for eliminating heterocyclic compounds such as carbazole (CA), benzothiophene (BT), and dibenzothiophene (DBT), which are among the most potent environmental pollutants [1]. Bacterial degradation pathways mainly involve the oxidation reactions such as angular dioxygenation of CA and dibenzofuran [2], lateral dioxygenation of DBT in Kodama pathway [3], and S-oxidation of DBT in 4S pathway [4]. A consequence of oxidation with oxygen (O_2_) is the formation of reactive oxygen species (ROS) [5]. Production of the ROS such as superoxide (O_2_^−^) and hydrogen peroxide (H_2_O_2_) determines the oxidative stress that threatens cells [6]. It has been reported that high levels of ROS were produced during the degradation of chlorinated biphenyls (CBs) [7], and were induced by 2-hydroxybiphenyl or 3-hydroxybiphenyl during biphenyl (BP) metabolism [8]. These ROS can damage DNA, proteins, and membranes [6], all of which are very crucial to bacteria living. It has also been reported that ROS could inhibit bacterial cell separation [8]. Hence, reacting quickly enough to ward off ROS attack is important for the survival of bacteria. Thus, the toxicity in bacteria due to excessive ROS production might contribute to the recalcitrance and persistence of contaminants in the environment.

To avoid oxidative damage, organisms have set up several antioxidant defenses comprising antioxidant enzymes such as superoxide dismutase and catalase, as well as antioxidative compounds. Carotenoids, present in a wide variety of bacteria, algae, fungi, and plants, are the most prominent membrane-integrated antioxidants [9]. They may play important roles in protecting cells against damaging radicals [10], while also changing membrane fluidity and proton permeability [11], [12]. Carotenoids could even affect the aging process of *Podospora anserina*, wherein over-expression of phytoene synthase and lycopene cyclase would prolong its life span by up to 31% [13]. The biosynthetic pathways of carotenoids and the corresponding genes have been elucidated in various organisms [14], [15]. Generally, C_40_ carotenoids are biosynthesized from geranylgeranyl pyrophosphate (GGPP) formed by GGPP synthase (CrtE). The head-to-head condensation of two molecules of GGPP by phytoene synthase (CrtB) forms phytoene. Subsequent dehydrogenation reactions catalyzed by phytoene desaturase (CrtI) introduce four double bonds into phytoene to produce red-colored lycopene. Cyclization reactions at both ends of lycopene catalyzed by lycopene cyclase (CrtY) yield *β*-carotene. Furthermore, *β*-carotene hydroxylase (CrtZ) can introduce hydroxyl groups into the *β*-ionone rings of *β*-carotene, converting it to zeaxanthin.

The *Sphingomonas* genus was first proposed in 1990 because all strains of the genus possess outer membranes containing glycosphingolipids [16]. By 2001, it was subdivided into four genera: *Sphingomonas*, *Sphingobium*, *Novosphingobium*, and *Sphingopyxis*, all of which are generically called sphingomonads [17]. In recent years, the degradation ability of xenobiotic compounds, particularly aromatic hydrocarbons, is a particularly attractive research topic in sphingomonad biology [18]. Colonies of many sphingomonads are yellow-pigmented [19]. Most sphingomonads contain nostoxanthin [20], [21], [22], while some of them contain other carotenoids such as zeaxanthin from *Sphingomonas jaspsi* [23], astaxanthin from *Sphingomonas astaxanthinifaciens* [24], and *β*-carotene from *Sphingomonas* sp. [25]. We have previously isolated a *Sphingobium yanoikuyae* strain XLDN2-5, which is able to degrade CA and transform BT and DBT co-metabolically [2], [26]. Based on its genome sequence [27], we supposed a C_40_ carotenoid biosynthetic pathway for strain XLDN2-5. Because carotenoids are part of a cellular defense system against ROS, we first set out to explore the ability of these compounds to influence the biodegradation of heterocyclic compounds in strain XLDN2-5. In the present study, we describe the amplification, identification, and functional analysis of phytoene desaturase, lycopene cyclase, and *β*-carotene hydroxylase, all of which are involved in carotenogenesis. Moreover, we also focus on the analysis of carotenoids from this strain, and the relationship between the carotenoid biosynthesis and biodegradation of heterocyclic compounds in strain XLDN2-5.

## Results

### Sequence Analysis of Carotenogenic Genes

**Figure 1:**
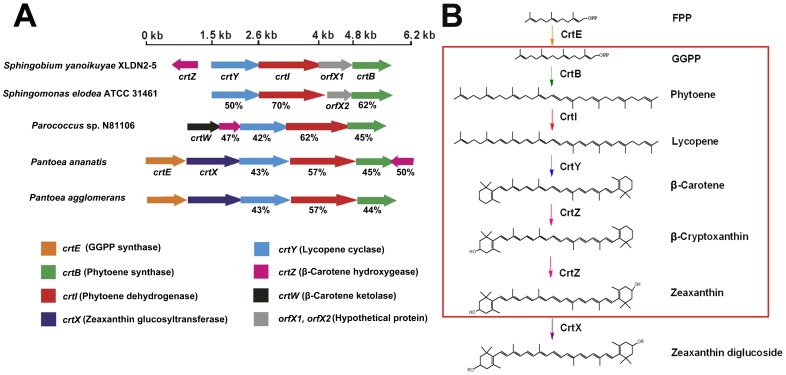
Carotenoid biosynthetic pathway and the genes involved in the biosynthesis of zeaxanthin in *Sphingobium yanoikuyae* XLDN2-5. (**A**) Genetic organization of the *crt* genes in strain XLDN2-5 compared to similar gene clusters from other bacteria. (**B**) The proposed carotenoid biosynthetic pathway in strain XLDN2-5 is inside the red box. CrtE: GGPP synthase (orange); CrtB: Phytoene synthase (green); CrtI: Phytoene dehydrogenase (red); CrtY: Lycopene cyclase (blue); CrtZ: *β*-Carotene hydroxygenase (magenta); CrtW: *β*-Carotene ketolase (black); CrtX: Zeaxanthin glucosyltransferase (purple); orfX1 and orfX2: No predicted function (grey). The percent of numbers represents the amino acid (aa) identities of related enzymes.

Using basic local alignment search tool (BLAST) analysis of the genome sequence of *Sphingobium yanoikuyae* XLDN2-5, several genes, designated as *crtE*, *crtB*, *crtI*, *crtY*, and *crtZ*, showed sequence homologies to GGPP synthase, phytoene synthase, phytoene dehydrogenase, lycopene cyclase, and *β*-carotene hydroxylase, respectively. It is well known that the carotenoid synthetic genes in many bacteria locate at the chromosomes as gene clusters [28], [29], [30]. However, these genes in strain XLDN2-5 are scattered, except for *crtY*, *crtI*, and the putative *crtB* (Figure 1A). The stop codon of the *crtY* gene overlaps the start codon of the *crtI* gene, which might be indicative for transcriptional coupling. In other bacteria that possess the *crtYIB* cluster, i.e. *Sphingobium chlorphenolicum* L-1 (AEG49620.1 and AEG49621.1) [31], *Sphingobium japonicum* UT26S (BAI94994.1 and BAI94995.1) [32], *Paracoccus* sp. N81106 (D58420) [30], [33], and *Bradyrhizobium* sp. ORS278 (AF218415) [34], these two genes *crtY* and *crtI* also overlap in a similar fashion. The CrtI protein showed 70%, 62%, 57%, 44%, and 42% amino acid identities with phytoene dehydrogenase from *Sphingomonas elodea* ATCC 31461 (ADO33738.1), *Paracoccus haeundaensis* (AAY28420.1), *Pantoea ananatis* (YP_005198070.1), *Deinococcus radiodurans* R1 (NP_294585.1), and *Rhodobacter capsulatus* SB1003 (YP_003576851.1), respectively. CrtE, CrtB, CrtY and CrtZ from strain XLDN2-5 have 35%, 45%, 43% and 50% amino acid identities, respectively, with GGPP synthase, phytoene synthase, lycopene cyclase and *β*-carotene hydroxylase from *P. ananatis* (D90087). Two highly conserved regions were found in CrtI: the putative dinucleotide-binding motif (*βαβ* fold) in the N-terminal region and the “bacterial type *Phytoene desaturase* signature” [35], [36], [37] at the C-terminus. Thus, we propose a carotenoid biosynthetic pathway in *Sphingobium yanoikuyae* XLDN2-5, as shown in the red frame of Figure 1B.

### Identification of Carotenoids

**Figure 2:**
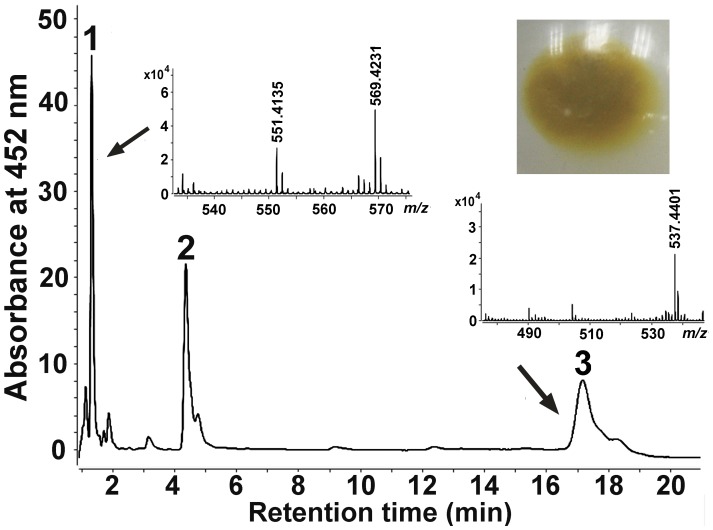
HPLC analysis and positive-ion APCI-MS spectra of the carotenoids produced by *Sphingobium yanoikuyae* XLDN2-5. The following carotenoids were identified: peak 1 was identified as zeaxanthin ([M+H]^+^ at *m/z* = 569.4231, and [M+H-H_2_O]^+^ at *m/z* = 551.4135); peak 2 was identified as *β*-cryptoxanthin; Peak 3 was identified as *β*-carotene ([M+H]^+^ at *m/z* = 537.4401). Insets show cell pellets from strain XLDN2-5.

*Sphingobium yanoikuyae* XLDN2-5 is a yellow-pigmented bacterium. To determine if the proposed carotenoid biosynthetic pathway was functional, we identified the yellow compounds produced by strain XLDN2-5. Using high-performance liquid chromatography (HPLC) with a diode array detector coupled with an atmospheric pressure chemical ionization mass spectrometer (HPLC-MS), we analyzed the pigments extracted from strain XLDN2-5 and found that there were three peaks having absorption maxima in the range of 400–500 nm, which were supposed to be carotenoids due to an extensive conjugated polyene chain (Figure S1). As shown in Figure 2, Peak 1 [peak absorption, UV-Vis λ_max_ of 452 nm and 478 nm; retention time, 1.3 min] has a characteristic fragment ion peak at *m/z* 569.4231 [M+H]^+^ with a dehydrated ion peak at *m/z* 551.4135 [M+H-H_2_O]^+^. This product was identified as zeaxanthin, as it shared identical chromatographic and spectral characteristics with *Escherichia coli*-derived zeaxanthin containing plasmid pACCAR25ΔcrtX [retention time, 1.3 min (Figure S2A); characteristic fragment ion peaks at *m/z* 569.4269 [M+H]^+^ and 551.4182 [M+H-H_2_O]^+^ (Figure S2B). Peak 3 [peak absorption, UV-Vis λ_max_ of 454 nm and 480 nm; retention time, 17.3 min] was identified as *β*-carotene (*m/z* 537.4401 [M+H]^+^), according to the retention time and *m/z* of the commercial authentic sample *β*-carotene (Sigma) (17.3 min; 537.4453 [M+H]^+^) (Figure S3A, S3B). Furthermore, we identified peak 2 was *β*-cryptoxanthin based on its peak absorption and molecular ion peak at *m/z* 552.5295 [M] and the characteristic fragment ion peak at m/z 535.4757 [M+H-H_2_O]^+^ (Figure S4). Therefore, we conclude that *Sphingobium yanoikuyae* XLDN2-5 accumulates zeaxanthin, which is synthesized from *β*-carotene through *β*-cryptoxanthin.

### Functional Identification of *crtY, crtI*, and *crtZ*

The genes, *crtY*, *crtI* and *crtZ*, found in strain XLDN2-5 were also expressed in *E. coli* DH5α. Plasmid pUcrtYI carrying *crtY* and *crtI* was introduced into *E. coli* DH5α carrying plasmid pACCRT-EB [30], which accumulates phytoene. In addition, the pUcrtZ plasmid carrying *crtZ* was introduced into *E. coli* DH5α carrying plasmid pACCAR16ΔcrtX [33], which accumulates *β*-carotene. Commercial *β*-carotene (Sigma) and zeaxanthin accumulated in *E. coli* DH5α carrying plasmid pACCAR25ΔcrtX [38] were used as standards. The resultant *E. coli* transformants were cultivated and harvested. Carotenoids accumulated in these cells were extracted with acetone and analyzed by HPLC and HPLC-MS.

**Figure 3:**
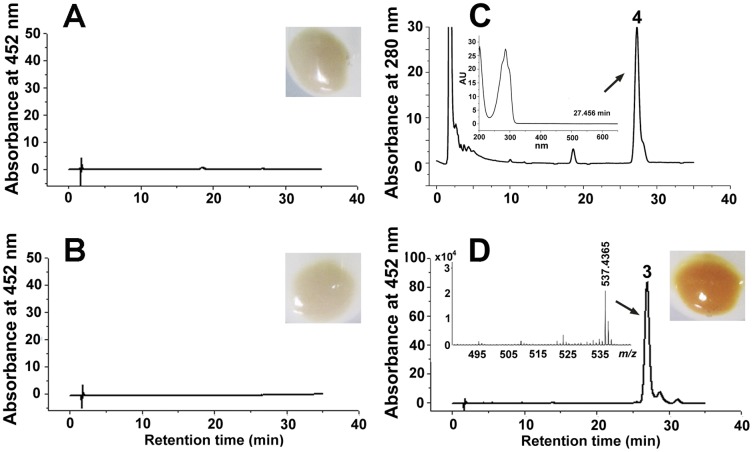
Pigmentation, HPLC analysis and positive-ion APCI-MS spectra of the carotenoids accumulated in *E. coli* for the functional identification of *crtY* and *crtI*. Detection at 452 nm: (**A**) *E. coli* containing plasmid pUcrtYI. (**B**) *E. coli* containing plasmid pACCRT-EB. (**D**) *E. coli* containing pACCRT-EB and pUcrtYI. Detection at 280 nm: (**C**) *E. coli* containing plasmid pACCRT-EB. Insets show cell pellets from *E. coli* transformants with different plasmids. Peak 3, *β*-carotene ([M+H]^+^ at *m/z* = 537.4365). Peak 4, phytoene. The absorption spectrum of peak 4 is shown in the inset in the profile of panel (C).

The *E. coli* strain carrying plasmid pUcrtYI (Figure 3A) or pACCRT-EB (Figure 3B) remained colorless, and no *β*-carotene was detected at 452 nm. Phytoene ([M+H]^+^ at *m/z* = 545.5027, Figure S5) was detected at 280 nm in the *E. coli* strain carrying plasmid pACCRT-EB (Figure 3C), which is the substrate of phytoene desaturase (CrtI). When plasmid pUcrtYI was introduced into the *E. coli* with pACCRT-EB, the cell pellet showed an orange pigmentation (Figure 3D), which was identified as *β*-carotene on the basis of its retention time at 26 min and *m/z* (537.4365 [M+H]^+^ and the commercial authentic sample (retention time, 26 min; *m/z* 537.4453 [M+H]^+^) (Figure S3B, S3C). These results indicate that the hypothetical gene *crtI* encodes the phytoene desaturase, which catalyzes lycopene synthesis from phytoene. The hypothetical gene *crtY* encodes lycopene cyclase, which catalyzes both cyclization steps required for the production of *β*-carotene from lycopene.

**Figure 4:**
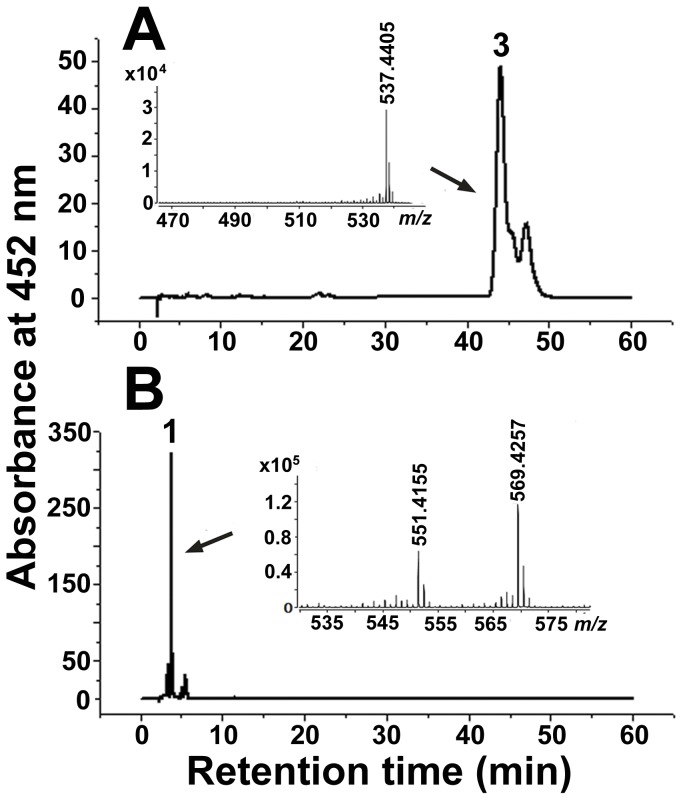
HPLC analysis and positive-ion APCI-MS spectra of the carotenoids accumulated in *E. coli* for the functional identification of *crtZ* (Detected at 452 nm). (**A**) *E. coli* containing pACCAR16ΔcrtX and pUC19. (**B**) *E. coli* containing pACCAR16ΔcrtX and pUcrtZ. Peak 1, zeaxanthin ([M+H]^+^ at *m/z* = 569.4257, and [M+H-H_2_O]^+^ at *m/z* = 551.4155). Peak 3, *β*-carotene ([M+H]^+^ at *m/z* = 537.4405).

The control strain, carrying pACCAR16ΔcrtX and pUC19, synthesized *β*-carotene (retention time, 43 min; *m/z* 537.4405 [M+H]^+^) (Figure 4A) in accordance with the commercial authentic sample (retention time, 43 min; *m/z* 537.4453 [M+H]^+^) (Figure S3B, S3D), while another control strain, carrying pACCAR25ΔcrtX, synthesized zeaxanthin (retention time, 4 min; a characteristic fragment ion peak at *m/z* 569.4269 [M+H]^+^ with a dehydrated ion peak at *m/z* 551.4182 [M+H-H_2_O]^+^) (Figure S2B, S2C). When plasmid pUcrtZ was introduced into the *E. coli* carrying pACCAR16ΔcrtX, the resultant recombinant strain produced predominantly zeaxanthin (retention time, 4 min; a characteristic fragment ion peak at *m/z* 569.4257 [M+H]^+^ with a dehydrated ion peak at *m/z* 551.4155 [M+H-H_2_O]^+^) (Figure 4B) from *β*-carotene. These results indicate that the hypothetical gene *crtZ* encodes *β*-carotene hydroxylase that catalyzes the synthesis of zeaxanthin from *β*-carotene.

### Effects of Heterocycles on Carotenoid Biosynthesis

**Figure 5:**
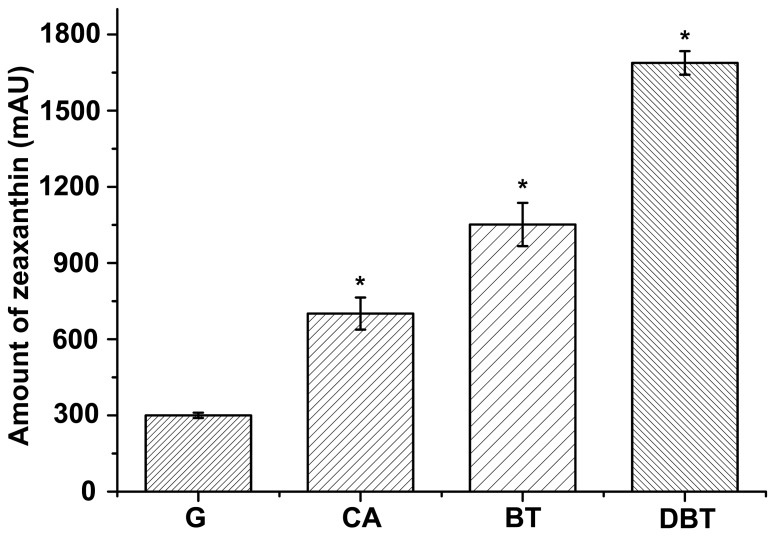
Amount of zeaxanthin accumulated in *Sphingobium yanoikuyae* XLDN2-5. Amount of zeaxanthin accumulated in strain XLDN2-5 in the absence (control, G: glucose and NH_4_Cl) or in the presence of heterocycles (CA: biodegradation of carbazole; BT: co-metabolism of CA and BT; DBT: co-metabolism of CA and DBT). Values represent the means of three independent experiments; the error bars represent standard deviations (*, p<0.01).

To characterize carotenoid biosynthesis in strain XLDN2-5, we investigated the amount of zeaxanthin produced by equal amounts of bacteria cell pellets during the degradation of CA and the transformation of BT or DBT. We used zeaxanthin obtained from strain XLDN2-5 as the blank control, which had been grown with glucose as the sole source of carbon. As shown in Figure 5, when the wild-type strain was grown using CA as the sole source of carbon, nitrogen and energy, the amount of zeaxanthin was >2-fold more than the blank control (p<0.01). Compared to the blank control, an obvious difference was also found in the co-metabolism of CA and BT or DBT. During the co-metabolism of CA and BT, the amount of zeaxanthin increased up to 380% (p<0.01). Furthermore, the accumulation of zeaxanthin in strain XLDN2-5 was significantly enhanced (up to 550%) during the co-metabolic transformation of CA and DBT (p<0.01). Therefore, the amount of zeaxanthin in strain XLDN2-5 was significantly enhanced during the biodegradation of heterocycles (CA < CA + BT < CA + DBT), as we expected.

### Analysis of Intracellular ROS

**Figure 6:**
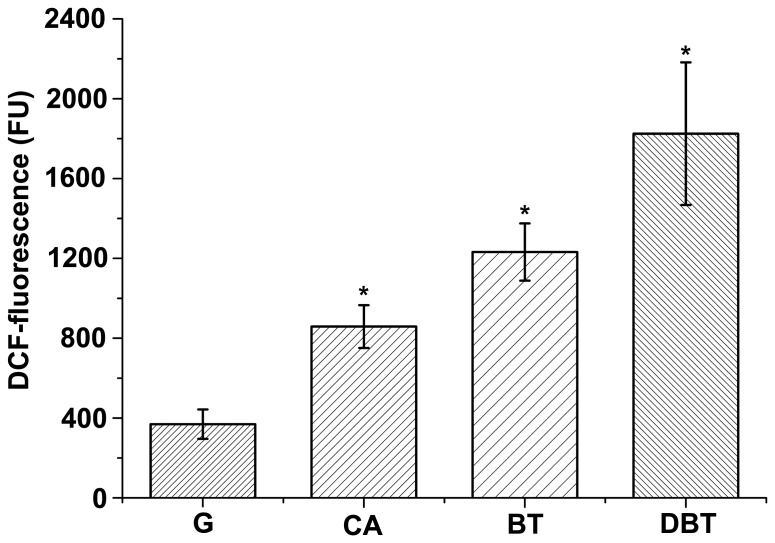
Determination of intracellular ROS by DCF fluorescence analysis. Determination of intracellular ROS with DCF-fluorescence in *Sphingobium yanoikuyae* XLDN2-5 in the absence (control, G: glucose and NH_4_Cl) or in the presence of heterocycles (CA: biodegradation of CA; BT: co-metabolism of CA and BT; DBT: co-metabolism of CA and DBT). Values represent the means of three independent experiments; the error bars represent standard deviations (*, p<0.01).

As carotenoids play important roles in protecting cells from oxidative damage, it is interesting to determine whether bacterial cells are stressed during the degradation of heterocycles. We measured the H_2_O_2_ levels in living bacterial cells with or without heterocycles (CA, BT, and DBT) by using the oxidative stress-sensitive probe 2′,7′-dichlorodihydrofluorescein-diacetate (DCFH-DA) [39], [40]. As shown in Figure 6, cells of strain XLDN2-5 grown with glucose had a lower fluorescence background generated as a result of H_2_O_2_ production (less than 400 FU). During the biodegradation of heterocycles, the green fluorescence of 2′,7′-dichlorofluorescein (DCF), which is the oxidation product of DCFH-DA, was significantly enhanced in cells of strain XLDN2-5. Compared to strain XLDN2-5 grown with glucose, the resultant DCF-fluorescence almost doubled with CA degradation (p<0.01), whereas 2-fold or nearly 4-fold increase was seen with the co-metabolism of CA and BT or DBT (p<0.01), respectively. These results provide clear evidence that excessive H_2_O_2_ are generated in bacterial cells during the degradation of heterocycles, and that bacterial cells were exposed to much more oxidative stress than the control cells were (strain XLDN2-5 grown with glucose). The observed increase in the H_2_O_2_ level in the bacterium was similar to the significant increase in the amount of zeaxanthin during the biodegradation of heterocycles (CA < CA + BT < CA + DBT).

### Transcript Analysis of Genes *crtI* and *crtZ*

**Figure 7:**
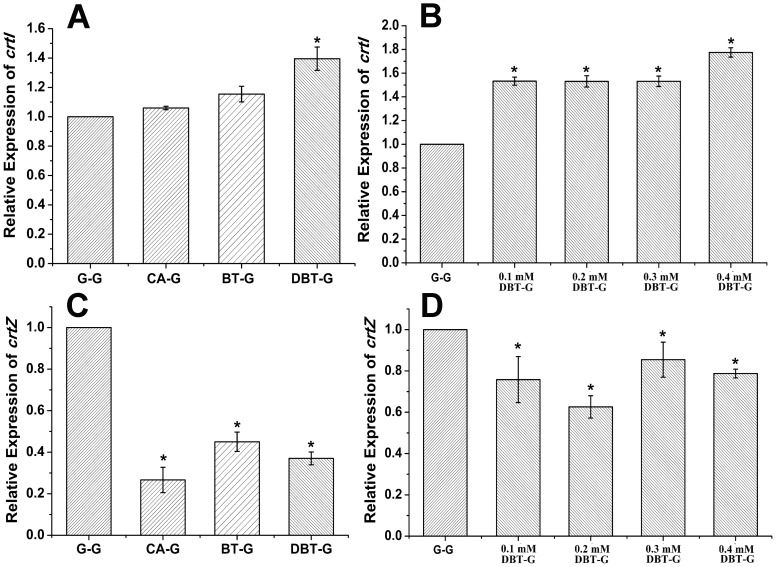
Relative expressions of *crtI* and *crtZ* determined by RT-qPCR. The values indicate changes relative to the levels of mRNA transcripts in *Sphingobium yanoikuyae* XLDN2-5 cultured in MSM with glucose and NH_4_Cl as the carbon and nitrogen sources, respectively. (**A**) Relative expression of *crtI* in bacterial cells, which were cultivated with glucose and NH_4_Cl, or CA, or CA and BT, or CA and DBT. (**B**) Relative expression of *crtI* in cells which were cultured in CA with the addition of 0.1 mM, 0.2 mM, 0.3 mM and 0.4 mM DBT. (**C**) Relative expression of *crtZ* in cells, which were cultivated with glucose and NH_4_Cl, or CA, or CA and BT, or CA and DBT. (**D**) Relative expression of *crtZ* in cells which were cultured by CA with the addition of 0.1 mM, 0.2 mM, 0.3 mM and 0.4 mM DBT. Values represent the means of four independent experiments; the error bars represent standard deviations (*, p<0.01).

To further explore the features of carotenoid biosynthesis underlying the heterocyclic compound degradation in *Sphingobium yanoikuyae* XLDN2-5, transcriptional levels of genes *crtI* and *crtZ* were determined using RT-qPCR (Figure 7). Strain XLDN2-5 was cultured in mineral salt medium (MSM) with various carbon and nitrogen sources. The values in Figure 7 indicated that any changes corresponded to the transcript levels in control bacterial cells that had been maintained in glucose and ammonium chloride (NH_4_Cl). As shown in Figure 7A, the mRNA content of *crtI* was affected by CA degradation, and transformation of BT or DBT, which changed in accordance with the amounts of zeaxanthin porduced. Around 6% and 15% increases in *crtI* transcription (p>0.01) were noted during the degradation of CA and transformation of BT, respectively. The most pronounced transcriptional rise in *crtI*, by about 38%, was observed with the co-metabolism of CA and DBT (p<0.01). When cells were cultured in CA by the addition of 0.1 mM, 0.2 mM and 0.3 mM DBT, all the transcriptional levels of *crtI* showed a 50% increase. Moreover, the transcriptional level of *crtI* increased by about 80% (p<0.01) with 0.4 mM DBT (Figure 7B). On the other hand, the transcriptional level of *crtZ* decreased relative to the control experiment, and no obvious regular change was observed for the *crtZ* transcripts with the degradation of the heterocycles (Figure 7C, 7D). The resultant higher enzyme levels of CrtI and lower enzyme levels of CrtZ may ensure the rapid accumulation of *β*-carotene, the precursor of zeaxanthin, which ultimately leads to the increased production of zeaxanthin.

## Discussion

Starting from the genome sequence of XLDN2-5 [27], several genes involved in the biosynthesis of carotenoids were identified and characterized; the proteins encoded by these genes shared low identities of amino acid sequences with corresponding enzymes from other species. Based on the results, a possible carotenoid biosynthetic pathway in *Sphingobium yanoikuyae* XLDN2-5 is illustrated in Figure 1B, wherein zeaxanthin is synthesized from *β*-carotene through *β*-cryptoxanthin. Previous studies showed that the carotenoid synthetic genes in many bacteria cluster together, particularly in those bacteria that carry a tightly linked *crtYIB* locus (Figure 1A) [28], [29], [30], and are under transcriptional co-regulation [41]. However, most of the carotenoid synthetic genes in strain XLDN2-5 locate separately from each other – except *crtY*, *crtI*, and the putative *crtB* (Figure 1A) – in a manner similar to the organization of genes involved in catabolic pathways in sphingomonads [42]. The comparative genomic research has revealed that horizontal gene transfer (HGT) is a major evolutionary pattern in microbial carotenoid biosynthesis [12]. Based on the organization of carotenoid synthetic genes in strain XLDN2-5 and transcriptional analysis above, we conclude that *crtZ* locates separately from the *crtBIY* cluster, and has a different pattern. This might be attributed to a distinct time course for *crtZ* genomic integration (as a result of HGT) as compared to the tightly linked *crtY* and *crtI* genes. Alternatively, the genome of strain XLDN2-5 might have undergone different DNA rearrangements responding to various environmental cues.

Bacterial degradation of heterocyclic compounds could generate several oxidation products. In this study, it is obvious that strain XLDN2-5 produced much more H_2_O_2_ during the co-metabolism of CA and BT or DBT than the control (Figure 6). Singlet oxygen or hydroxyl radicals might be generated from H_2_O_2_ by the Mallet reaction [43] or Fenton reaction [6]. Therefore, the ROS formation mechanism in the bacterium is likely associated with the degradation. Recent studies showed that 30–35% DBT co-metabolized by strain XLDN2-5 was transformed into DBT sulfoxide (DBTO) and DBT sulfone (DBTO_2_) [2]. Sulfoxides and sulfones could also be formed during the microbial transformation of BTs [26]. The potent oxidant O(^3^P) might be produced during the direct photolysis of DBTO [44]; O(^3^P) or other oxidant species were reportedly produced during the formation of benzo[*b*]naphtha[1,2-*d*]thiophene from benzothiophene-1-oxide [26]. Moreover, high levels of ROS were also induced in other bacteria during the degradation of CBs [7] and BP [8]. It was reported that the leakage of electrons from electron transport chain (ETC) is the main source of ROS in most cell types [45]. Alternatively, ROS might also be produced due to the membrane stress, resulting from the exposure of heterocycles, i. e., a compromised ETC. Nevertheless, all of these ROS could impose much stress on cells, and it is reasonable to suggest that the ROS-generated toxicity during the degradation may contribute to the recalcitrance and persistence of the heterocycles in the environment. As a possible survival adaptation mechanism, strain XLDN2-5 accumulated massive amounts of zeaxanthin; in addition, the transcription rate of the gene (*crtI*) encoding phytoene desaturase, one of the key enzymes for carotenoid biosynthesis [46], also increased significantly. Zeaxanthin, one of the carotenoids, is a stronger ROS scavenger [47] and offers better protection in liposomes than *β*-carotene [48]. Therefore, when bacterial cells are exposed to the oxidative stress as a result of cellular metabolism, zeaxanthin accumulated in the bacterial cell membrane might quench the ROS, thus reducing their lethal effects.

For the transformation of BT or DBT, strain XLDN2-5 needs to grow on CA to induce the expression of BT- or DBT-degrading enzymes, and the BT or DBT molecules should enter bacterial cells to be degraded. Consequently, their degradation rates may depend on the structure of the bacterial cell membrane. The polar zeaxanthin molecule spans lipid bilayer, interacting with acyl lipid chains via van Der Waals forces, thereby causing the fluid phase of cell membrane to turn rigid [49]. Moreover, polar carotenoids may reduce oxygen diffusion through the cell membrane [50]. Increases in the polar zeaxanthin production by strain XLDN2-5 might decrease the membrane fluidity during the biodegradation; enhance the diffusion barrier of the membrane to balance the generation and quenching of ROS; thus ultimately protect this bacterium from oxidative damage, resulting in an improved strain XLDN2-5 survival rate and more efficient biodegradation. Additionally, CA and BT have log *P*_o-w_ values of about 3, and DBT has a log *P*_o-w_ value of about 4 [51], [52]. Interaction of the molecules with the cell membrane might affect the membrane fluidity by disruption of lipid packing [53], [54], [55]. It was reported that carotenoids accumulated in the membrane might also reduce the space into which heterocycles can insert and decrease the extent of interaction [56]. Therefore, an alternative hypothesis might be that the carotenoid, zeaxanthin, responds to the membrane fluidity, which should be confirmed in future investigations.

In summary, *Sphingobium yanoikuyae* XLDN2-5 could produce zeaxanthin from *β*-carotene through *β*-cryptoxanthin via a carotenoid synthetic pathway. Our results suggest that carotenoids might play a positive role in the degradation of heterocycles. In addition to being an important cellular defense against oxidative stress resulting from the degradation of heterocycles, carotenoids may modulate membrane fluidity and proton permeability to control the degradation rates. This study implies a new research topic which would shed light on the advance of bioremediation or biocatalysis.

## Materials and Methods

### Materials

*β*-Carotene (≥99% purity), DCFH-DA, CA, BT, and DBT were purchased from Sigma-Aldrich (St. Louis, MO, USA). Isopropyl-*β*-d-1-thiogalactopyranoside (IPTG) was from Merck (Darmstadt, Germany). All other commercially available chemicals were of analytical grade or were chromatographically pure.

### Bacterial Strains, Plasmids, and Culture Conditions

The bacterial strains and plasmids used in this study are listed in Table 1. *Sphingobium yanoikuyae* XLDN2-5 was cultivated in MSM as previously described [2], and CA was added in the form of a 200-mM filter-sterilized stock solution prepared in dimethyl sulfoxide. In experiments dealing with mixtures of CA, BT and DBT, 2 mM CA, 0.3 mM BT and 0.2 mM DBT were added to 50 mL of MSM. *E. coli* DH5α was used as the host strain in cloning experiments. *E. coli* strains for molecular manipulation were grown in lysogeny broth (LB) medium containing 50 mg·L^−1^ ampicillin, at 37°C, with shaking at 200 rpm. For pigment analysis, *E. coli* transformants carrying two distinct plasmids were separately cultured in LB medium containing ampicillin (100 µg·mL^−1^), chloramphenicol (40 µg·mL^−1^), and 0.5 mM IPTG, in the dark at 28°C.

**Table 1:** Bacterial strains and plasmids used in this study.

Strain and plasmid	Description	Source or reference
**Strain**		
*Sphingobium yanoikuyae* XLDN2-5	Wild-type (CCTCC M205093)	Laboratory stock
*E. coli* DH5α	F^-^, φ80d*lacZ*ΔM15, Δ(*lacZYA-argF*)U169, *deoR*, *recA*1, *endA*1, *hsdR*17(r_k_^-^, m_k_^+^), *phoA*,*supE*44, λ^−^, *thi*-1, *gyrA*96, *relA*1	TransGen
*E. coli* DH5α_YI_	DH5α containing plasmid pUcrtYI	This work
*E. coli* DH5α_EB_	DH5α containing plasmid pACCRT-EB, accumulating phytoene	This work
*E. coli* DH5α_EBYI_	DH5α containing plasmid pUcrtYI and pACCRT-EB	This work
*E. coli* DH5α_16+Z_	DH5α containing plasmid pACCR16ΔcrtX and pUcrtZ	This work
*E. coli* DH5α_16+pUC_	DH5α containing plasmid pACCR16ΔcrtX and pUC19	This work
*E. coli* DH5α_16_	DH5α containing plasmid pACCR16ΔcrtX, accumulating *β*-carotene	This work
*E. coli* DH5α_25_	DH5α containing plasmid pACCR25ΔcrtX, accumulating zeaxanthin	This work
**Plasmid**		
pUC19	Cloning vector (Ap^r^), Amp^r^ *lacZ*, pMB9 replicon, M13IG	Takara
pUcrtZ	pUC19 with 519bp *Xba*I-*Eco*RI fragment containing the *crtZ* gene of *Sphingobium* *yanoikuyae* XLDN2-5 (Ap^r^)	This work
pUcrtYI	pUC19 with 2630bp *Hind*III-*Xba*I fragment containing *crtY* and *crtI* genes of*Sphingobium yanoikuyae* XLDN2-5 (Ap^r^)	This work
pACCRT-EB	pACYC184 containing *crtE* and *crtB* genes of *P. ananatis* (Cm^r^)	[30]
pACCR16ΔcrtX	pACYC184 containing *crtE*, *crtB*, *crtI*, *crtY* genes of *P. ananatis* (Cm^r^)	[33]
pACCR25ΔcrtX	pACYC184 containing *crtE*, *crtB*, *crtI*, *crtY* and *crtZ* genes of *P. ananatis* (Cm^r^)	[38]

### Construction of Plasmids for Expression of Carotenogenic Genes

The manipulation and transformation of DNA were performed according to standard procedures [57]. The lycopene cyclase and phytoene dehydrogenase genes (*crtY* and *crtI*), and the *β*-carotene hydroxylase gene (*crtZ*) were amplified using PCR involving the respective primers listed in Table S1. Total DNA from strain XLDN2-5 was used as a template during PCR amplification. The resultant 2,630-bp fragment containing the *crtY* and *crtI* genes was digested with *Hind*III-*Xba*I restriction enzymes, and cloned into the *Hind*III-*Xba*I sites of a pUC19 vector to construct plasmid pUcrtYI. The resultant 519-bp fragment containing the *crtZ* gene was then digested with *Xba*I-*EcoR*I restriction enzymes, and then cloned into the *Xba*I-*EcoR*I sites of pUC19 to construct plasmid pUcrtZ. Plasmid pUcrtYI was introduced into the phytoene producing *E. coli* DH5α containing pACCRT-EB [30]. Plasmid pUcrtZ was introduced into the *β*-carotene producing *E. coli* DH5α containing pACCAR16ΔcrtX [33]. Other *E. coli* DH5α transformants harboring different combinations of plasmids are listed in Table 1.

### Analysis of Carotenoids

Strain XLDN2-5 or *E. coli* DH5α transformants were cultured and induced as described above. Cells were harvested by centrifugation at 8,000 × *g*, and washed twice in distilled water. Carotenoids were extracted from cells in the dark by using acetone, and the supernatant was collected, filtered, and analyzed using an HPLC instrument (Agilent 1200 series, Hewlett-Packard, CA, USA) equipped with an Agilent Eclipse XDB-C18 column (4.6×150 mm or 4.6×250 mm, 5 µm). The column was eluted with methanol:2-propanol (80∶20) at a flow rate of 1 mL·min^−1^. The eluted fractions were detected at 452 or 280 nm by using a diode array detector. Commercial *β*-carotene (Sigma) and zeaxanthin obtained from *E. coli* DH5α carrying plasmids pACCAR25ΔcrtX [38] were used as standards. The carotenoid pigments were identified on the basis of retention time, features of absorption spectra, and comparison with standard compounds or reported data. The amount of zeaxanthin in strain XLDN2-5 was determined on the basis of its HPLC peak area at 452 nm. The HPLC-MS analysis was conducted by an Accurate-Mass Q-TOF LC/MS instrument (Agilent G6520A, Hewlett-Packard, USA) equipped with a Zorbax extend-C18 column (4.6 × 50 mm, 1.8 µm). Crude extract was eluted at a rate of 0.3·mL min^−1^ with gradient elution of solvent A (acetonitrile:methanol (0.1M ammonium acetate):dichloromethane, 71∶22:7, v:v:v) to 30% solvent B (20 mM ammonium acetate in acetonitrile). Mass spectra were monitored in the *m/z* range of 300–700.

### Detection of Reactive Oxygen Species

For the detection of intracellular ROS in strain XLDN2-5, cells were grown in 500 mL of MSM with 2 mM CA, or 2 mM CA and 0.3 mM BT, or 2 mM CA and 0.2 mM DBT, or 1 g·L^−1^ glucose and 0.5 g·L^−1^ NH_4_Cl (as a control). Cells were then collected and washed twice in 10 mL of 100 mM phosphate buffer (pH 7.4). The resultant cell pellets were then resuspended in the same buffer, adjusted to an optical density (OD_600_) of 1.0, and then incubated with 10 µM DCFH-DA (prepared from a 10-mM stock solution in ethanol) for 30 min at 37°C. The DCF (2′,7′-dichlorofluorescein) green fluorescence was measured using a Synergy 2 Multi-Mode Microplate Reader (Bio-Tek, Vermont, USA), and the measured fluorescence produced by cells was expressed in fluorescence units (FU).

### RNA Preparation and RT-qPCR

After cultivating strain XLDN2-5 in MSM supplemented with 2 mM CA at 30°C, cells of the culture (500 mL) were harvested by centrifugation at 5,000 × *g* for 5 min, and then washed twice in MSM. The washed cells were then suspended in 20 mL of MSM. The resultant cell suspension (5 mL) was incubated with 2 mM CA, or 2 mM CA and 0.3 mM BT, or 2 mM CA and 0.2 mM DBT, or 1 g·L^−1^ glucose and 0.5 g·L^−1^ NH_4_Cl (as a calibrator) to identify the relative expressions of *crtI* and *crtZ* with various combinations of heterocycles. To compare the relative expressions of *crtI* and *crtZ* with varied amounts of DBT, cells were cultured in CA with the addition of 0.1 mM, 0.2 mM, 0.3 mM and 0.4 mM DBT. After 2-h incubation with reciprocal shaking (200 rpm) at 30°C, cells were harvested and used for extraction of total RNA (TianGen Biotech, Beijing, China). Reverse transcription was performed using an iScript cDNA Synthesis Kit (Bio-Rad, Hemel Hempstead Corp., UK) in which 100 ng of total RNA was used as starting material.

RT-qPCR was performed using a CFX96 RT-qPCR Detection System (Bio-Rad). Amplifications were carried out using iQ™ SYBR Green Supermix (Bio-Rad) in a final volume of 25 µL with 0.3 µM specific primers and equal amounts of cDNA. The RT-qPCR primers are listed in Table 1. The 16S-rRNA gene of strain XLDN2-5 was set as the reference gene. Cycling conditions for the RT-qPCR reaction were as follows: 95°C for 2 min followed by 40 cycles at 95°C for 10 s, 55.7°C (for *crtI*) or 57°C (for *crtZ*) or 59°C (for 16S-rDNA) for 10 s, and 68°C for 15 s. Each sample was run in triplicate. All results were normalized to the internal 16S-rRNA standard a subsequently analyzed following the comparative methods of Livak [58] or Pfaffl [59].

## Supporting Information

Figure S1 **Absorption spectra for the major peaks in** **Figure 2** **from HPLC analysis of the carotenoids produced by** ***Sphingobium yanoikuyae*** **XLDN2-5.**
(PDF)

Figure S2 **HPLC analysis under different chromatographic conditions and positive-ion APCI-MS spectrum of zeaxanthin produced by** ***E. coli*** **containing pACCAR25ΔcrtX.** (A) The retention time of zeaxanthin was about 1.3 min for HPLC-MS analysis. The Zorbax extend-C18 column (4.6 × 50 mm, 1.8 µm) was eluted at a rate of 0.3 mL min^−1^ with gradient elution of solvent A (acetonitrile:methanol (0.1 M ammonium acetate):dichloromethane, 71∶22:7, v:v:v) to 30% solvent B (20 mM ammonium acetate in acetonitrile). (B) Positive-ion APCI-MS spectrum of zeaxanthin. (C) The retention time of zeaxanthin was about 4 min for the HPLC analysis. The Agilent Eclipse XDB-C18 column (4.6 × 250 mm, 5 µm) was eluted with methanol:2-propanol (80∶20) at a flow rate of 1 mL·min^−1^.
(PDF)

Figure S3 **HPLC analysis under different chromatographic conditions and positive-ion APCI-MS spectrum of commercial** ***β*** **-carotene (Sigma).** (A) The retention time of *β*-carotene was about 17.3 min for the HPLC-MS analysis. (B) Positive-ion APCI-MS spectrum of *β*-carotene. (C) The retention time of *β*-carotene was about 26 min for the HPLC analysis. The Agilent Eclipse XDB-C18 column (4.6 × 150 mm, 5 µm) was eluted with methanol:2-propanol (80∶20) at a flow rate of 1 mL·min^−1^. (D) The retention time of *β*-carotene was about 43 min for the HPLC analysis. The Agilent Eclipse XDB-C18 column (4.6 × 250 mm, 5 µm) was eluted with methanol:2-propanol (80∶20) at a flow rate of 1 mL·min^−1^.
(PDF)

Figure S4 **Positive-ion APCI-MS spectrum of peak 2 in** **Figure 2** **.** This peak was identified as *β*-cryptoxanthin based on its peak absorption and molecular ion peak at *m/z* 552.5295 [M] and the characteristic fragment ion peak at *m/z* 535.4757 [M+H-H_2_O]^+^.
(PDF)

Figure S5 **Positive-ion APCI-MS spectrum of phytoene produced by** ***E. coli*** **concomitantly with pACCRT-EB.** Peak 4 in Figure 3C was identified as phytoene based on its characteristic fragment ion peak at *m/z* 545.5027 [M+H]^+^.
(PDF)

Table S1 **Primers used in this study.**
(DOC)
