# Supplementary material for: Carotenoids Play a Positive Role in the Degradation of Heterocycles by Sphingobium yanoikuyae
Source: PLoS One. 2012 Jun 20;7(6):e39522. doi: 10.1371/journal.pone.0039522 (PMC3380023; doi:10.1371/journal.pone.0039522)
Supplement: Figure S1 — Absorption spectra for the major peaks in Figure 2 from HPLC analysis of the carotenoids produced by Sphingobium yanoikuyae XLDN2-5. (PDF) [file pone.0039522.s001.pdf]

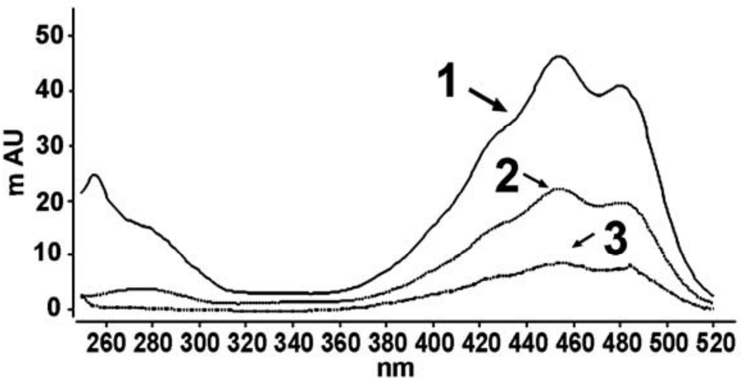


**Figure S1. Absorption spectra for the major peaks in Figure 2 from HPLC analysis of the carotenoids produced by *Sphingobium yanoikuyae* XLDN2-5.**
