# Supplementary material for: Carotenoids Play a Positive Role in the Degradation of Heterocycles by Sphingobium yanoikuyae
Source: PLoS One. 2012 Jun 20;7(6):e39522. doi: 10.1371/journal.pone.0039522 (PMC3380023; doi:10.1371/journal.pone.0039522)
Supplement: Figure S2 — HPLC analysis under different chromatographic conditions and positive-ion APCI-MS spectrum of zeaxanthin produced by E. coli containing pACCAR25ΔcrtX. (A) The retention time of zeaxanthin was about 1.3 min for HPLC-MS analysis. The Zorbax extend-C18 column (4.6 × 50 mm, 1.8 µm) was eluted at a rate of 0.3 mL min−1 with gradient elution of solvent A (acetonitrile:methanol (0.1 M ammonium acetate):dichloromethane, 71∶22:7, v:v:v) to 30% solvent B (20 mM ammonium acetate in acetonitrile). (B) Positive-ion APCI-MS spectrum of zeaxanthin. (C) The retention time of zeaxanthin was about 4 min for the HPLC analysis. The Agilent Eclipse XDB-C18 column (4.6 × 250 mm, 5 µm) was eluted with methanol:2-propanol (80∶20) at a flow rate of 1 mL·min−1. (PDF) [file pone.0039522.s002.pdf]

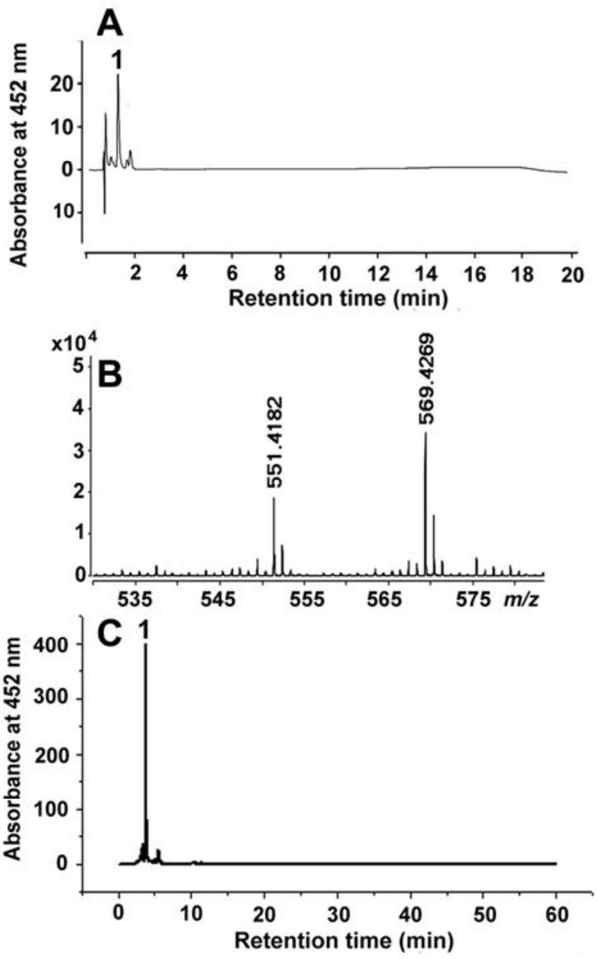

Figure S2. HPLC analysis under different chromatographic conditions and positive-ion APCI-MS spectrum of zeaxanthin produced by *E. coli* containing pACCAR25ΔcrtX. (A) The retention time of zeaxanthin was about 1.3 min for HPLC-MS analysis. The Zorbax extend-C18 column (4.6 × 50 mm, 1.8 μm) was eluted at a rate of 0.3 mL min^{-1} with gradient elution of solvent A (acetonitrile:methanol (0.1M ammonium acetate):dichloromethane, 71:22:7, v:v:v) to 30% solvent B (20 mM ammonium acetate in acetonitrile). (B) Positive-ion APCI-MS spectrum of zeaxanthin. (C) The retention time of zeaxanthin was about 4 min for the HPLC analysis. The Agilent Eclipse XDB-C18 column (4.6 × 250 mm, 5 μm) was eluted with methanol:2-propanol (80:20) at a flow rate of 1 mL·min^{-1}.
