# Supplementary material for: Carotenoids Play a Positive Role in the Degradation of Heterocycles by Sphingobium yanoikuyae
Source: PLoS One. 2012 Jun 20;7(6):e39522. doi: 10.1371/journal.pone.0039522 (PMC3380023; doi:10.1371/journal.pone.0039522)
Supplement: Figure S3 — HPLC analysis under different chromatographic conditions and positive-ion APCI-MS spectrum of commercial β -carotene (Sigma). (A) The retention time of β-carotene was about 17.3 min for the HPLC-MS analysis. (B) Positive-ion APCI-MS spectrum of β-carotene. (C) The retention time of β-carotene was about 26 min for the HPLC analysis. The Agilent Eclipse XDB-C18 column (4.6 × 150 mm, 5 µm) was eluted with methanol:2-propanol (80∶20) at a flow rate of 1 mL·min−1. (D) The retention time of β-carotene was about 43 min for the HPLC analysis. The Agilent Eclipse XDB-C18 column (4.6 × 250 mm, 5 µm) was eluted with methanol:2-propanol (80∶20) at a flow rate of 1 mL·min−1. (PDF) [file pone.0039522.s003.pdf]

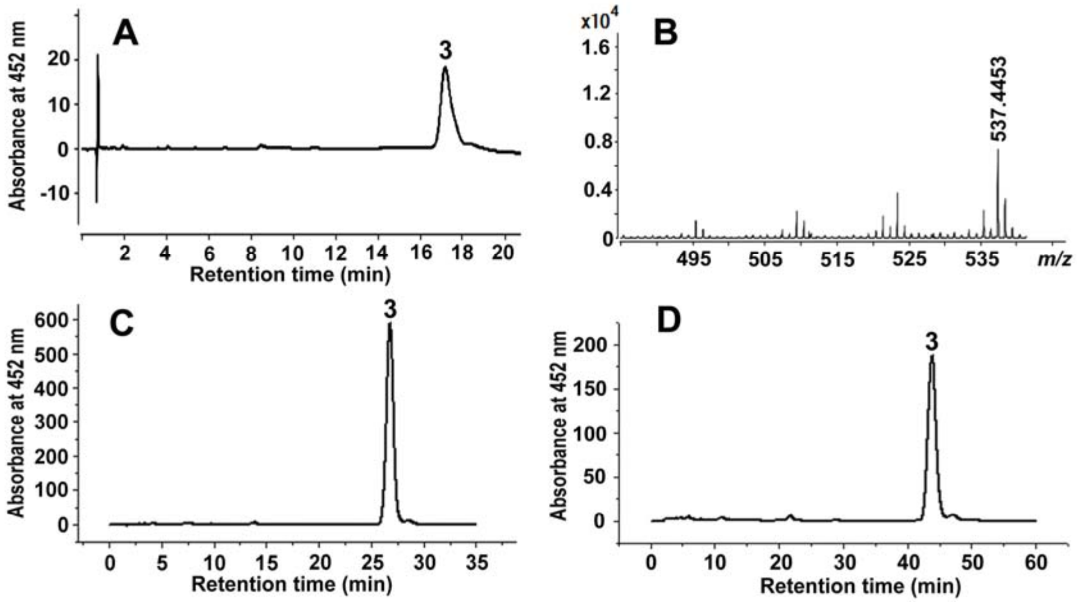

Figure S3. HPLC analysis under different chromatographic conditions and positive-ion APCI-MS spectrum of commercial *β*-carotene (Sigma). (A) The retention time of *β*-carotene was about 17.3 min for the HPLC-MS analysis. (B) Positive-ion APCI-MS spectrum of *β*-carotene. (C) The retention time of *β*-carotene was about 26 min for the HPLC analysis. The Agilent Eclipse XDB-C18 column (4.6 × 150 mm, 5 μm) was eluted with methanol:2-propanol (80:20) at a flow rate of 1 mL·min^{-1}. (D) The retention time of *β*-carotene was about 43 min for the HPLC analysis. The Agilent Eclipse XDB-C18 column (4.6 × 250 mm, 5 μm) was eluted with methanol:2-propanol (80:20) at a flow rate of 1 mL·min^{-1}.
