# Supplementary material for: Carotenoids Play a Positive Role in the Degradation of Heterocycles by Sphingobium yanoikuyae
Source: PLoS One. 2012 Jun 20;7(6):e39522. doi: 10.1371/journal.pone.0039522 (PMC3380023; doi:10.1371/journal.pone.0039522)
Supplement: Figure S4 — Positive-ion APCI-MS spectrum of peak 2 in Figure 2 . This peak was identified as β-cryptoxanthin based on its peak absorption and molecular ion peak at m/z 552.5295 [M] and the characteristic fragment ion peak at m/z 535.4757 [M+H-H2O]+. (PDF) [file pone.0039522.s004.pdf]

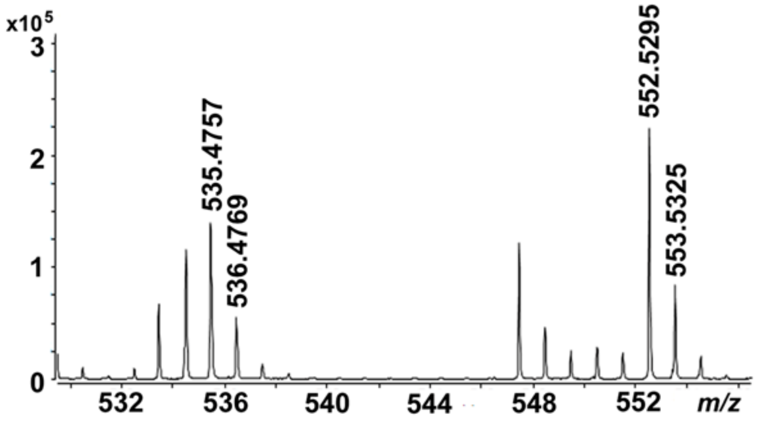

Figure S4. Positive-ion APCI-MS spectrum of peak 2 in Figure 2. This peak was identified as *β*-cryptoxanthin based on its peak absorption and molecular ion peak at *m/z* 552.5295 [M] and the characteristic fragment ion peak at *m/z* 535.4757 $[M+H-H_2O]^+$.
