# Supplementary material for: Carotenoids Play a Positive Role in the Degradation of Heterocycles by Sphingobium yanoikuyae
Source: PLoS One. 2012 Jun 20;7(6):e39522. doi: 10.1371/journal.pone.0039522 (PMC3380023; doi:10.1371/journal.pone.0039522)
Supplement: Figure S5 — Positive-ion APCI-MS spectrum of phytoene produced by E. coli concomitantly with pACCRT-EB. Peak 4 in Figure 3C was identified as phytoene based on its characteristic fragment ion peak at m/z 545.5027 [M+H]+. (PDF) [file pone.0039522.s005.pdf]

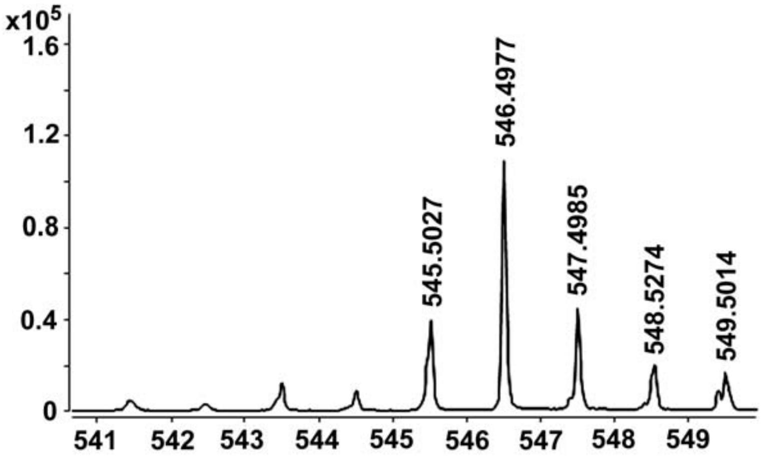

Figure S5. Positive-ion APCI-MS spectrum of phytoene produced by *E. coli* concomitantly with pACCRT-EB. Peak 4 in Figure 3C was identified as phytoene based on its characteristic fragment ion peak at *m/z* 545.5027 $[M+H]^+$.
