# Supplementary material for: Carotenoids Play a Positive Role in the Degradation of Heterocycles by Sphingobium yanoikuyae
Source: PLoS One. 2012 Jun 20;7(6):e39522. doi: 10.1371/journal.pone.0039522 (PMC3380023; doi:10.1371/journal.pone.0039522)
Supplement: Table S1 — Primers used in this study. (DOC) [file pone.0039522.s006.doc]

**Table S1.** **Primers used in this study.**

| **Primer** | **Sequence** ^a)^ | **Tarrget gene** |
| --- | --- | --- |
| **Primer for gene cloning** | | |
| *crtY*-F^b)^ | 5′-AAAAAAGCTTCTAAGGAGGTGTTCATatgGCTGATCTGGAATGCGA-3′ | *crtY* and *crtI* |
| *crtI*-R^c)^ | 5′-AAAATCTAGAtcaAGCCATGTCCTGCA  GCA-3′ |
| *crtZ*-F | 5′-AAAATCTAGACTAAGGAGGTGTTCATatgCCTCCAATCGCCCTCCTG-3′ | *crtZ* |
| *crtZ*-R | 5′-AAAAGAATTCctaGTCCCGCGTGCCGGGCT-3′ |
| **Primer for RT-qPCR transcript quantification** | | |
| 16S-qPCR-F | 5′-AGCGTTGTTCGGAATTAC-3′ | 16S*-*rRNA |
| 16S-qPCR-R | 5′-CACCTCTACACTCGGAAT-3′ |
| *crtI*-qPCR-F | 5′-CACCATATGATCCTGTTC-3′ | *crtI* |
| *crtI*-qPCR-R | 5′-AGATAGAGCGAGAAATCC-3′ |
| *crtZ*-qPCR-F | 5′-ATGCCTCCAATCGCCCTC-3′ | *crtZ* |
| *crtZ*-qPCR-R | 5′-CAGCGGTGCATCACATAG-3′ |

^a)^ Restriction enzyme sites are underlined.

^b)^ "F" stands for the forward primer.

^c)^ "R" stands for the reverse primer.
